# A novel telomerase-derived peptide GV1001-mediated inhibition of angiogenesis: Regulation of VEGF/VEGFR-2 signaling pathways

**DOI:** 10.1016/j.tranon.2022.101546

**Published:** 2022-09-29

**Authors:** Jae Hyeon Kim, Young-Rak Cho, Eun-Kyung Ahn, Sunho Kim, Surim Han, Sung Joon Kim, Gyu-Un Bae, Joa Sub Oh, Dong-Wan Seo

**Affiliations:** a Department of Pharmacy, College of Pharmacy, Dankook University, Cheonan 31116, Republic of Korea; b Biocenter, Gyeonggido Business & Science Accelerator, Suwon 16229, Republic of Korea; c Department of Pharmacy, College of Pharmacy, Sookmyung Women's University, Seoul 04310, Republic of Korea

**Keywords:** GV1001, Telomerase-derived peptide, Angiogenesis, VEGF, Non-small cell lung cancer

## Abstract

GV1001, a human telomerase reverse transcriptase catalytic subunit-derived 16-mer peptide, has been developed as a novel anticancer vaccine against various cancers including pancreatic cancer. In the current study, we demonstrate the regulatory roles and mechanisms of GV1001 in endothelial cell responses *in vitro* and microvessel sprouting *ex vivo*. GV1001 markedly inhibits vascular endothelial growth factor-A (VEGF-A)-stimulated endothelial cell permeability, proliferation, migration, invasion, tube formation as well as microvessel outgrowth from rat aortic rings. These anti-angiogenic effects of GV1001 were associated with the inhibition of VEGF-A/VEGFR-2 signaling pathways, redistribution of vascular endothelial-cadherin to cell–cell contacts, and down-regulation of VEGFR-2 and matrix metalloproteinase-2. Furthermore, GV1001 suppresses the proliferation and invasion of non-small cell lung cancer cells, and the release of VEGF from the cells, suggesting the regulatory role of GV1001 in tumor-derived angiogenesis as well as cancer cell growth and progression. Collectively, our study reports the pharmacological potential of GV1001 in the regulation of angiogenesis, and warrants further evaluation and development of GV1001 as a promising therapeutic agent for a variety of angiogenesis-related diseases including cancer.

## Highlights

• GV1001, a human telomerase-derived peptide, inhibits endothelial cell proliferation, migration, invasion, tube formation and microvessel sprouting.
• GV1001 regulates VEGF-A-stimulated signaling network components including FAK, Src, MEK, ERK and AKT pathways.
• GV1001 suppresses VEGF-A-induced expression of VEGFR-2 and MMP-2.

## Introduction

Angiogenesis plays pivotal roles in the pathogenesis and progression of diseases such as cancer, rheumatoid arthritis, ischemic heart disease, macular degeneration and diabetic retinopathy as well as normal physiological development and maintenance [1], [2], [3], [4]. Vascular endothelial growth factor-A (VEGF-A), one of the main angiogenesis regulators, and VEGF receptor-2 (VEGFR-2) signal transduction pathways have been widely appreciated as the key therapeutic targets for a broad range of angiogenesis-related diseases [5], [6], [7], [8], [9]. The potent role of VEGF in angiogenesis is evidenced by the development and clinical use of bevacizumab, an anti-VEGF antibody, for therapy in various types of human cancer including colorectal cancer, lung cancer, renal cancer and ovarian cancer as well as ocular diseases [10,11]. However, lots of drugs targeting VEGF and VEGF/VEGFR-2 signaling pathways in the clinic often result in disease relapse and progression associated with drug resistance. Therefore, in-depth understanding of cell signaling networks may provide potential therapeutic targets and strategies for improving clinical outcomes without serious side effects.

The human telomerase reverse transcriptase (hTERT), the catalytic subunit of telomerase holoenzyme, is responsible for telomere maintenance and chromosomal stability. Given that hTERT is highly expressed in cancer cells and stem cells, hTERT has been appreciated as a promising therapeutic target for the treatment of cancer [12], [13], [14], [15]. GV1001, a 16-mer peptide derived from the active site of hTERT, is composed of amino acid residues between E^611^ and K^626^ of the hTERT (EARPALLTSRLRFIPK), and generates a wide variety of hTERT-specific T responses including CD4^+^ and CD8^+^ T cells for cancer regression and improved survival [16], [17], [18].

Many investigations have demonstrated that GV1001 possesses anti-tumor activity against a variety of cancers such as pancreatic cancer, cutaneous T cell lymphoma, B-cell chronic lymphocytic leukemia, cutaneous melanoma, non-small cell lung cancer (NSCLC), renal cancer and prostate cancer [19], [20], [21], [22], [23]. Although GV1001 has received substantial attention as a potential cancer vaccine for inducing T cell responses, several results in clinical trials have been marginal or disappointing [24], [25], [26], [27]. Overcoming therapeutic limitation of GV1001 requires further understanding of the molecular mechanisms and targets underlying the pathogenesis of cancer growth and progression. In addition to anti-tumor activity, GV1001 has been reported to exert various biological activities such as anti-inflammatory, anti-oxidative and anti-viral activities [28], [29], [30], [31].

However, the effects and molecular mechanisms of GV1001 in angiogenesis which play important roles in various pathological conditions such as cancer, ocular and inflammatory diseases have not been investigated in detail. The current study aims to investigate the effects and signaling pathways of GV1001 on endothelial cell fates, which is essential for angiogenic responses.

## Materials and methods

### Cell culture conditions

Human umbilical vein endothelial cells (HUVECs) from Lonza (Walkersville, MD, USA) were cultured in EGM-2® BulletKit media and used in the passage ranges 4–6 for all experiments, according to the manufacturer's instructions (Lonza) [32]. Human non-small cell lung cancer cells (NSCLC: A549, H1299) from the American Type Culture Collection (Manassas, VA, USA) were grown in 10% fetal bovine serum (FBS)-Dulbecco's modified Eagle's medium (DMEM) (Hyclone Laboratories, Logan, UT, USA).

### Reagents

GV1001, a human telomerase-derived 16-mer peptide, was provided by GemVax-KAEL (Seongnam, Republic of Korea). The following agents were obtained from commercial sources: vascular endothelial growth factor-A 165 (Merck Millipore, Billerica, MA, USA); anti-phospho-VEGFR-2 (Y1175), anti-phospho-MEK (S217/S221), anti-MEK, anti-phospho-Src (Y416), anti-Src, anti-phospho-p70^S6K^ (T421/S424), anti-phospho-Akt (S473), anti-phospho-ERK (T202/Y204), anti-phospho-pRb (S780), and anti-phospho-pRb (S807/S811) (Cell Signaling Technology, Beverly, MA, USA); fibroblast growth factor-2 (FGF-2), anti-phosphotyrosine, anti-phospho-FAK(Y397), anti-FAK, anti-β-catenin, and anti-p120-catenin (BD Biosciences, Bedford, MA, USA); anti-vascular endothelial (VE)-cadherin, anti-VEGFR-2, anti-p70^S6K^, anti-Akt, anti-ERK, anti-Cdk2, anti-Cdk4, anti-cyclin D, anti-cyclin E, anti-p27^kip1^, anti-actin antibodies, and mouse and rabbit IgG-horseradish peroxidase conjugates (Santa Cruz Biotechnology, Santa Cruz, CA, USA); goat anti-mouse IgG-Alexa Fluor 488 conjugate (Thermo Fisher Scientific Co., Waltham, MA, USA).

### Cell permeability

HUVECs, plated on gelatin-coated fluorescence blocking polyethylene terephthalate (PET) membrane inserts (FluoroBlok™ Insert, 3.0 μm pore size, BD biosciences) in 24-well plates (8×10^4^ cells/well), were cultured in EGM-2® BulletKit media until they had reached confluence. After serum starvation in endothelial cell basal medium-2 (EBM-2, Lonza) for 1 h, FITC-dextran (MW 40 kDa, Sigma-Aldrich Co., St. Louis, MO, USA) was added to the upper compartment, and the cells were treated with GV1001 (0.05–5 μM) for 30 min, followed by VEGF-A (10 ng/mL) stimulation for 30 min. The fluorescence was measured at 480/520 nm with Synergy Mx plate reader (BioTek Instruments, Winooski, VT, USA).

### Immunofluorescence microscopy

Quiescent HUVECs, plated on gelatin-coated coverslips in 12-well plates (BD Biosciences), were treated with GV1001 (5 μM) for 30 min, followed by VEGF-A (10 ng/mL) stimulation for 30 min. After cell fixation and permeabilization, cells were blocked with 5% BSA-PBS and incubated with primary antibodies, followed by Alexa Fluor 488-conjugated secondary antibodies (Thermo Fisher Scientific Co.). Images were observed using a Carl Zeiss Microscope (Axio Imager.M2) and AxioVision Rel. 4.8 software (Zeiss Co., Gottingen, Germany). Fluorescence intensities were quantified using NIH ImageJ version 1.51j8 software.

### Cell viability and proliferation

HUVECs, plated on 6-well plates (1×10^5^ cells/well), were serum-starved for 14 h in EBM-2 and treated with GV1001 (0.05–5 μM) for 30 min, followed by VEGF-A (10 ng/mL) stimulation for 24 h. In some experiments, human non-small cell lung cancer cells (A549 and H1299), plated on 6-well plates (5×10^4^ cells/well), were serum-starved for 24 h in basal DMEM and treated with GV1001 (0.05–5 μM) for 30 min, followed by 10% FBS stimulation for 24 h. Cell proliferation and viability were determined as described previously. Results from triplicate determinations (mean ± standard deviation) are presented as the fold-increase of the untreated controls, the number of cells per culture or the percentage of live cells of total cell counts.

### Cell cycle analysis

Quiescent HUVECs were treated with GV1001 (0.05–5 μM) for 30 min, followed by VEGF-A (10 ng/mL) stimulation for 24 h. Cells were fixed with ice-cold 70% ethyl alcohol, stained with Muse™ cell cycle reagent, and then analyzed by a Muse™ cell analyzer (Merck Millipore) [33].

### Cell migration assay

A single wound was created in the center of confluent HUVEC monolayer by a sterile pipette tip. After serum starvation in EBM-2 for 2 h, cells were treated with GV1001 (0.05–5 μM) for 30 min, followed by VEGF-A (10 ng/mL) stimulation for 16 h. Following fixation with methanol, cells were stained with 0.04% Giemsa solution (Sigma-Aldrich Co.). The migration of cells across a wound field gap was quantified as previously described [34].

### Cell invasion assay

Transwell invasion assay was performed as previously described [35]. HUVECs or cancer cells, plated on Matrigel® (BD Biosciences)-coated transwell inserts (Costar, 6.5 mm diameter insert, 8 μm pore size) (Corning Inc., Corning, NY, USA), were serum-starved for 2 h and treated with GV1001 (0.05–5 μM) for 30 min, followed by VEGF-A (10 ng/mL) or 10% FBS stimulation for 18 h. After fixation with methanol, invasive cells were stained with 0.04% Giemsa solution and quantified from six different fields using x200 objective magnification.

### Zymogram analysis

Effect of GV1001 on matrix metalloproteinase (MMP) activities was measured by zymography [36]. Aliquots of conditioned media collected from HUVECs treated with GV1001 (5 μM) and VEGF-A (10 ng/mL) for 16 h were subjected to SDS-PAGE on gels containing 0.1% gelatin (Sigma-Aldrich Co.) as a substrate. After electrophoresis, the gels were soaked in 2.5% Triton X-100 for 1 h to remove SDS, and then incubated in developing buffer (pH 7.5) containing 50 mM Tris-HCl, 150 mM NaCl and 10 mM CaCl_2_ for 16 h at 37 °C. The gels were stained with 0.5% Coomassie brilliant blue R-250 in 30% methyl alcohol-10% acetic acid for 3 h, and then destained with 30% methyl alcohol-10% acetic acid. Gelatinolytic MMP activities were detected as transparent bands against a dark blue background of stained gelatin. Band intensities were quantified using NIH ImageJ version 1.51j8 software.

### RNA isolation and reverse transcriptase-polymerase chain reaction (RT-PCR)

Total RNA was isolated using PureHelix™ Total RNA Purification kit (Nanohelix Co., Daejeon, Republic of Korea). RNA purity and concentration were determined using a NanoDrop™ 2000 spectrophotometer (Thermo Fisher Scientific Co.). RNA (1 μg) was used as a template for RT-mediated PCR using 1st Strand cDNA Synthesis kit (BioAssay Co., Daejeon, Republic of Korea). Primer sets for MMP-2 were forward 5′-GCTCAGATCCGTGGTGAGAT-3′ and reverse 5′-GGTGCTGGCTGAGTAGATCC-3′; primer sets for VEGFR-2 were forward 5′-TGCCTACCTCACCTGTTTCCT-3′ and reverse 5′-TACACGGTGGTGTCTGTGTCA-3′; primer sets for glyceraldehydes-3-phosphate dehydrogenase (GAPDH) were forward 5′-GAAGGTGAAGGTCGGAGTC-3′ and reverse 5′-GAAGATGGTGATGGGATTTC-3′; and the primer sets for VEGF were forward 5′-TCGGGCCTCCGAAACCATGA-3′ and reverse 5′-CCTGGTGAGAGATCTGGTTC-3′.

### Immunoprecipitation and western blot analysis

Quiescent HUVECs were treated with GV1001 (5 μΜ) for 30 min, followed by VEGF-A (10 ng/mL) stimulation for the indicated time points. Cells were lysed by incubation in 50 mM Tris-HCl (pH 7.4), 150 mM NaCl, 1% Triton X-100, 0.5 μg/mL leupeptin, 1 μg/mL pepstatin A, 10 μg/mL aprotinin, 100 μg/mL 4-(2-aminoethyl)benzenesulfonyl fluoride, 1 mM EDTA, 1 mM sodium orthovanadate, 25 mM sodium fluoride, 80 mM β-glycerophosphate and 10% glycerol for 30 min at 4 °C. Cell lysates were subjected to immunoprecipitation and Western blot as previously described [37]. Band intensities were quantified using NIH ImageJ version 1.51j8 software.

### Tube formation assays

After serum starvation in EBM-2 for 2 h, cells (4×10^4^ cells/mL) were plated on Matrigel®-coated 24-well plates and treated with GV1001 (0.05–5 μM) for 30 min, followed by VEGF-A (10 ng/mL) for 6 h. Formation of capillary-like structures was examined using an Olympus CKX41 inverted microscope (CAchN 10/0.25php objective) and ToupTek Toupview software (version x86, 3.5.563, Hangzhou ToupTek Photonics Co., Zhejiang, P. R. China) .

### Rat aortic ring assay

Eight- to nine-week old male Sprague-Dawley rats (250 ± 10 g) were purchased from RaonBio Inc. (Yongin, Republic of Korea). The animal experiments were conducted in accordance with the institutional guidelines. The experimental procedures were approved by the Institutional Animal Care and Use Committee at Dankook University (Cheonan, Republic of Korea). Thoracic aortic ring segments embedded in Matrigel® were treated with GV1001 (5 μM) for 30 min, followed by VEGF-A (500 ng/mL) for 3 days and then incubated with fresh GV1001 and VEGF-A every other day, and photographed on the 7th day using x40 objective magnification. The area of microvessel sprouting was quantified using Adobe PhotoShop software.

### VEGF enzyme-linked immunosorbent assay (ELISA)

Quiescent NSCLC cells were treated with GV1001 (5 μM) for 30 min, followed by 10% FBS stimulation for 48 h. Using a VEGF ELISA kit (R&D Systems, Minneapolis, MN, USA) VEGF levels in the conditioned media was measured at 450 nm with Synergy Mx plate reader (BioTek Instruments).

### Statistical analysis

Statistical analysis was performed using Student's *t*-test, and was based on at least three different experiments. *P* values < 0.05 were considered to be statistically significant.

## Results

### GV1001 inhibits VEGF-A-stimulated endothelial cell permeability through suppression of VE-cadherin phosphorylation and induction of VE-cadherin localization at cell-cell contacts

**Fig. 1:**
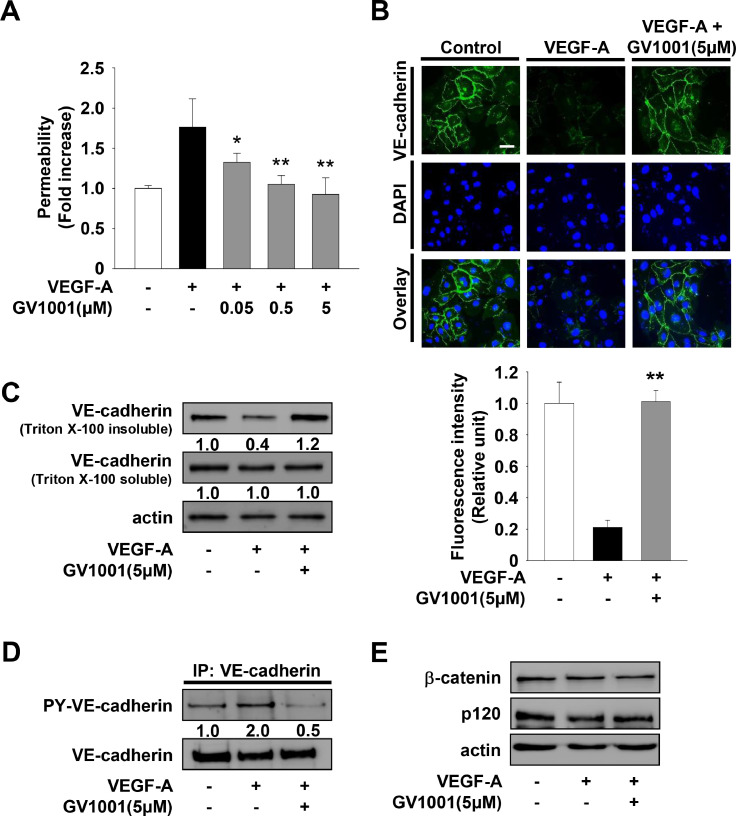
GV1001 inhibits VEGF-A-stimulated endothelial permeability through the regulation of VE-cadherin phosphorylation and distribution. Quiescent cells were treated with GV1001 (0.05–5 μM) for 30 min, followed by VEGF-A (10 ng/mL) stimulation for 30 min. (A) Results from three independent experiments (mean ± SD) are presented as the fold-increase of FITC-dextran permeability in untreated controls. Statistical significance is indicated (*p < 0.05, **p < 0.01, compared with VEGF-A-treated cells). (B - E) Cells were treated with GV1001 (5 μM) for 30 min, followed by VEGF-A stimulation for 30 min as described in panel A. (B) Distribution of VE-cadherin was determined as described in Materials and methods. DNA was stained with 4′,6-diamidino-2-phenylindole (DAPI). Scale bar represents 10 μm. (C) Distribution of VE-cadherin was assessed by Triton X-100 solubility. Integrated density values for VE-cadherin in Triton X-100 insoluble fraction were normalized to untreated controls. (D) Anti-VE-cadherin immunoprecipitates (IP) were Western-blotted with anti-phosphotyrosine or anti-VE-cadherin antibodies. (E) Cell lysates were Western-blotted with anti-β-catenin, anti-p120 or anti-actin antibodies. Results are representative of at least three independent experiments.

Localization of vascular endothelial (VE)-cadherin at adherens junctions is closely associated with endothelial barrier function, leading to maintenance of tissue integrity [38]. VEGF-A-mediated endothelial cell activation induces the loss of VE-cadherin at cell–cell contacts and subsequent endothelial cell permeability, the earliest event of the angiogenic responses [39], [40], [41]. The roles and functions of VE-cadherin can be assessed by the levels of VE-cadherin detectable at cell surfaces or in the Triton-insoluble cell fraction, together with those of tyrosine phosphorylated VE-cadherin in the Triton-soluble fraction [7,42]. We first examined the effect of GV1001 on endothelial cell permeability in HUVECs. GV1001 treatment inhibited VEGF-A-stimulated cell permeability in a dose-dependent manner (Fig. 1A). To determine whether VE-cadherin plays a role in GV1001 regulation of endothelial permeability, we next examined the ability of GV1001 to modulate VE-cadherin phosphorylation and distribution in VEGF-A-treated HUVECs. GV1001 treatment blocked the VEGF-A-induced loss of VE-cadherin and β-catenin from cell surfaces and in the Triton-insoluble fraction to levels observed in unstimulated controls (Fig. 1B, C and Supplementary Fig. 1). Consistent with this observation, GV1001 inhibited VEGF-A-induced phosphorylation of VE-cadherin in the Triton-soluble fraction (Fig. 1D). The levels of VE-cadherin, β-catenin and p120-catenin showed little or no change following VEGF-A stimulation in the presence or absence of GV1001 (Fig. 1C and E). Collectively, these observations demonstrate that GV1001-mediated inhibition of VEGF-A-induced cell permeability are closely associated with suppression of VE-cadherin tyrosine phosphorylation and subsequent stabilization of VE-cadherin at cell–cell contacts.

### GV1001 inhibits VEGF-A-stimulated cell proliferation through modulation of cell cycle-related proteins

**Fig. 2:**
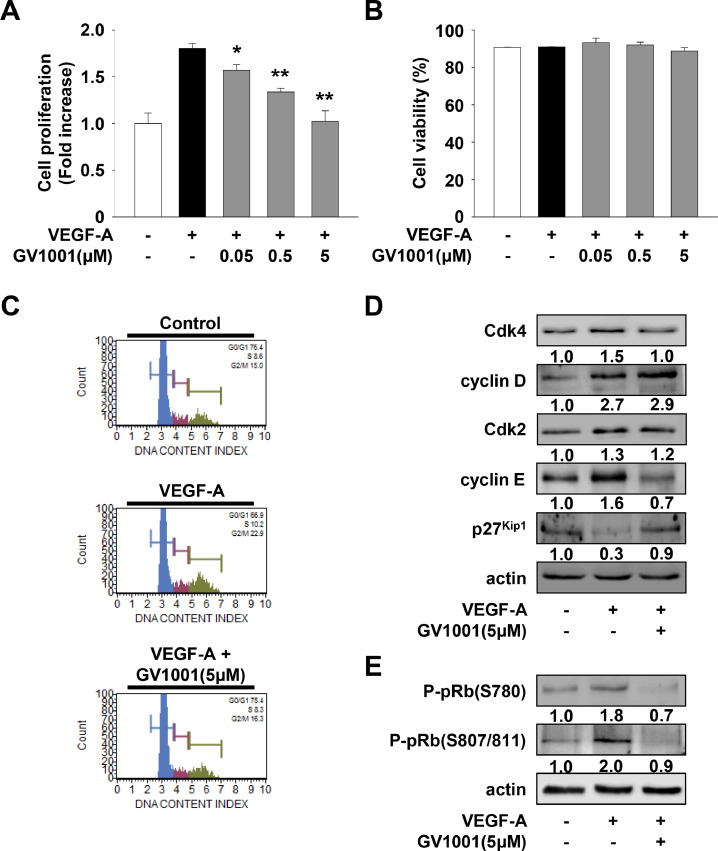
GV1001 exerts anti-proliferative activity in VEGF-A-treated HUVECs. Quiescent cells were treated with GV1001 (0.05–5 μM) for 30 min, followed by VEGF-A (10 ng/mL) stimulation for 24 h. (A) Cell proliferation and (B) viability results from three independent experiments (mean ± SD) are presented as the fold-increase of untreated controls or the percentage of live cells of total cell counts. Statistical significance is indicated (*p < 0.05, **p < 0.01, compared with VEGF-A-treated cells). (C) Cell cycle and (D, E) Western blot analyses were performed as described in Materials and methods. Cells were treated with GV1001 (5 μM) for 30 min, followed by VEGF-A stimulation for 24 h as described in panel A. Results are representative of at least three independent experiments.

We next analyzed the effect of GV1001 on the proliferation of HUVECs. GV1001 treatment dose-dependently inhibited VEGF-A-stimulated cell proliferation (Fig. 2A) and did not alter cell viability (Fig. 2B) at the highest concentration used in the current study, demonstrating the potential efficacy of GV1001 in regulating endothelial cell proliferation with little or no cytotoxicity. In addition, GV1001 inhibited the proliferation in FGF-2 stimulated cells (Supplementary Fig. 2A), similar to that in VEGF-A-treated cells (Fig. 2A). Alone GV1001 treatment without VEGF-A or FGF-2 did not affect the basal proliferation (data not shown). Given the ability of GV1001 to inhibit VEGF-A-stimulated cell proliferation, we examined the effect of GV1001 on the cell cycle distribution by measuring DNA content (Fig. 2C). GV1001 markedly inhibited VEGF-A-induced changes in the phase distribution of cell cycle to the levels observed in untreated controls. These findings indicate that GV1001 induces G_1_ cell cycle arrest, which is well correlated with suppression of cell proliferation (Fig. 2A). We next examined the changes of cell cycle-related proteins in GV1001-treated HUVECs. As shown in Fig. 2D and E, GV1001 markedly suppressed VEGF-A-induced phosphorylation of pRb by down-regulation of cyclin-dependent kinase 4 (Cdk4) and cyclin E, and by up-regulation of p27^Kip1^. These data show the anti-proliferative activity of GV1001 by blocking the G_1_-S phase transition.

### GV1001 inhibits VEGF-A-stimulated cell migration, invasion and tube formation *in vitro*, and microvessel sprouting *ex vivo*

**Fig. 3:**
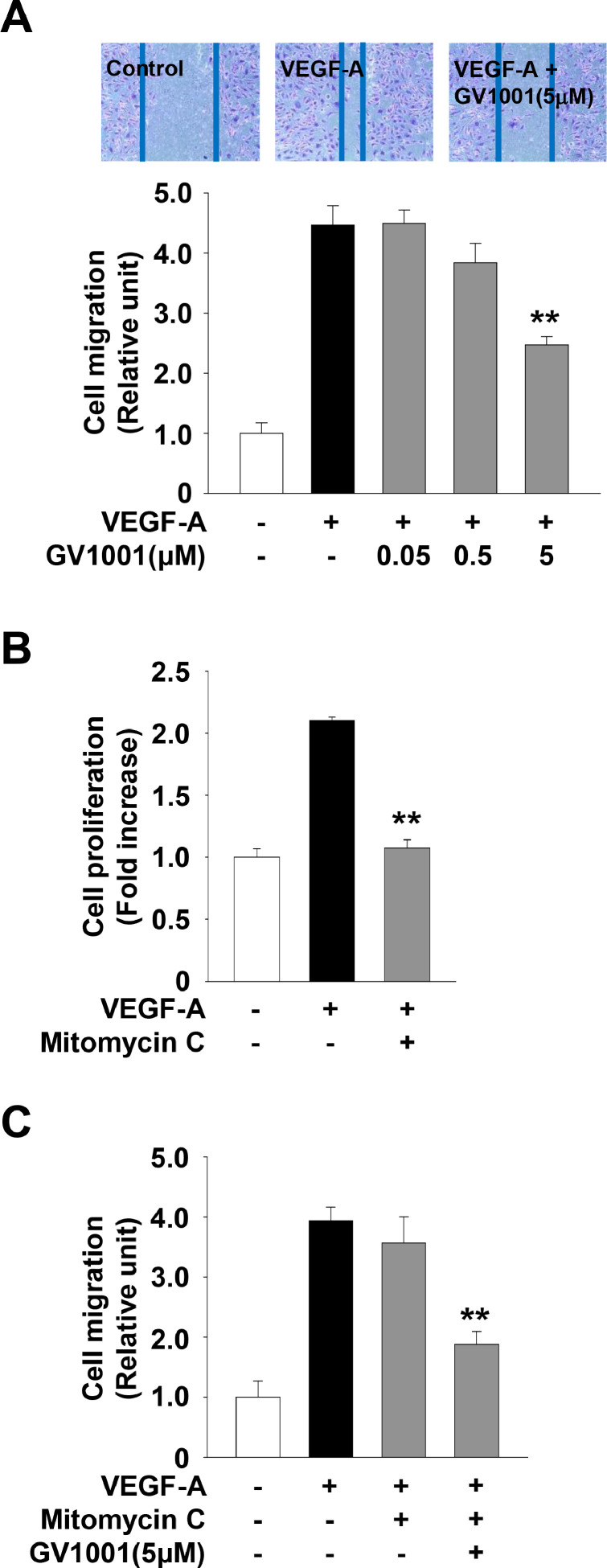
GV1001 possesses anti-migratory activity in VEGF-A-treated HUVECs. (A) Quiescent cells were treated with GV1001 (0.05–5 μM) for 30 min, followed by VEGF-A (10 ng/mL) stimulation for 16 h. Results from at least three independent experiments (mean ± SD) are presented as the fold-increase of untreated controls. (B) Quiescent cells were treated with mitomycin C (5 μg/mL) for 30 min, followed by VEGF-A (10 ng/mL) stimulation for 24 h. (C) Quiescent cells were treated with GV1001 (5 μM) for 30 min in the presence of mitomycin C (5 μg/mL), followed by VEGF-A-stimulation for 16 h. Statistical significance is indicated (**p < 0.01, compared with VEGF-A-treated cells).

GV1001 treatment inhibited VEGF-A-stimulated cell migration in a dose-dependent manner (Fig. 3A). However, these observations raise the possibility that GV1001-mediated inhibition of cell proliferation (Fig. 2A) might contribute to anti-migratory activity of GV1001. Thus, we examined the changes in anti-migratory effect of GV1001 in the presence of mitomycin C (5 μg/ml) to inhibit cell cycle progression. As shown in Fig. 3B and C, mitomycin C markedly suppressed the proliferation in VEGF-A-treated HUVECs, but did not alter the migratory response to VEGF-A stimulation. GV1001 treatment similarly inhibited VEGF-A-stimulated cell migration in the presence or absence of mitomycin C, demonstrating that the anti-migratory activity of GV1001 might not be a result from inhibition of cell proliferation.

**Fig. 4:**
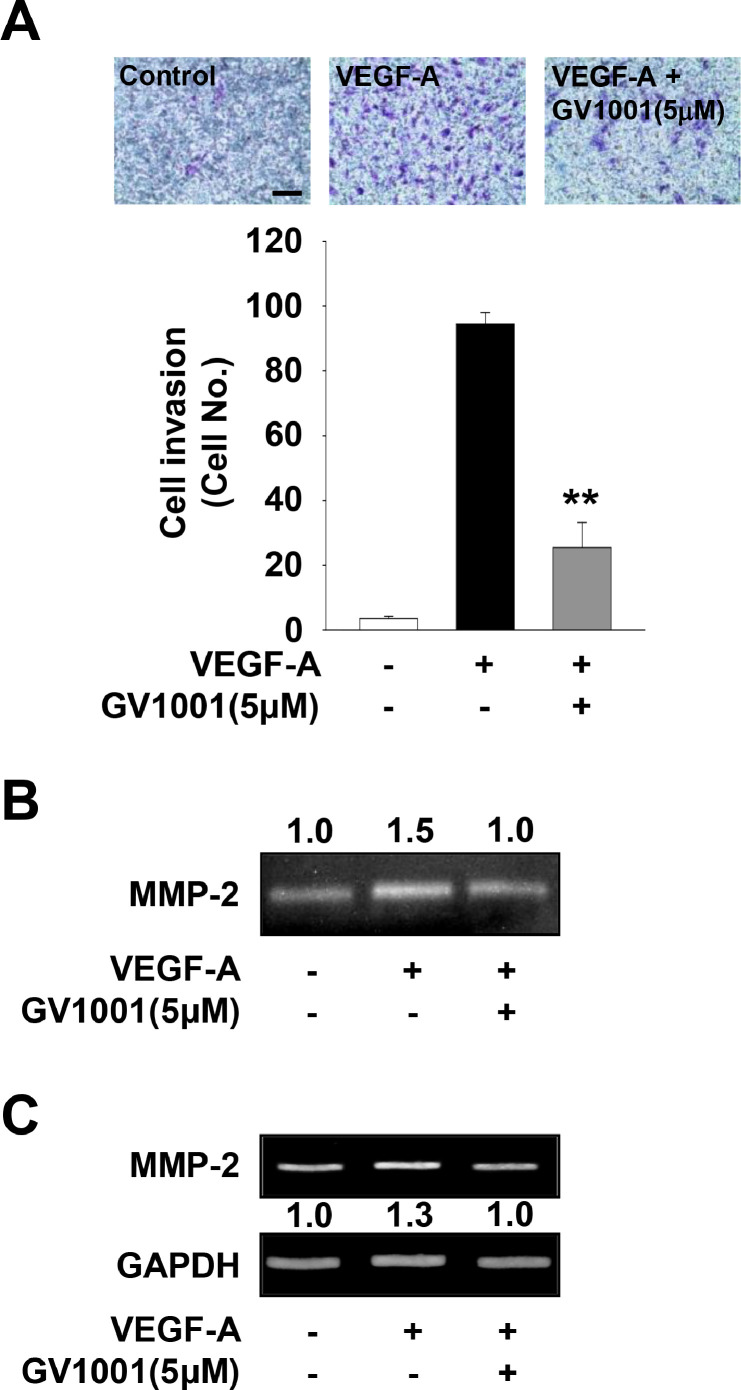
GV1001 shows anti-invasive activity in VEGF-A-treated HUVECs. Quiescent cells were treated with GV1001 (5 μM) for 30 min, followed by VEGF-A (10 ng/mL) stimulation for 18 h. (A) Results from at least three independent experiments (mean ± SD) are presented as the numbers of invasive cells. Scale bar represents 100 μm. Statistical significance is indicated (**p < 0.01, compared with VEGF-A-treated cells). (B) Gelatin zymogram analysis was performed using conditioned media from cell culture treated as in panel A. Zymogram gel loading was normalized to total protein concentration. (C) Expression of MMP-2 was determined by RT-PCR analysis. Results are representative of at least three independent experiments.

**Fig. 5:**
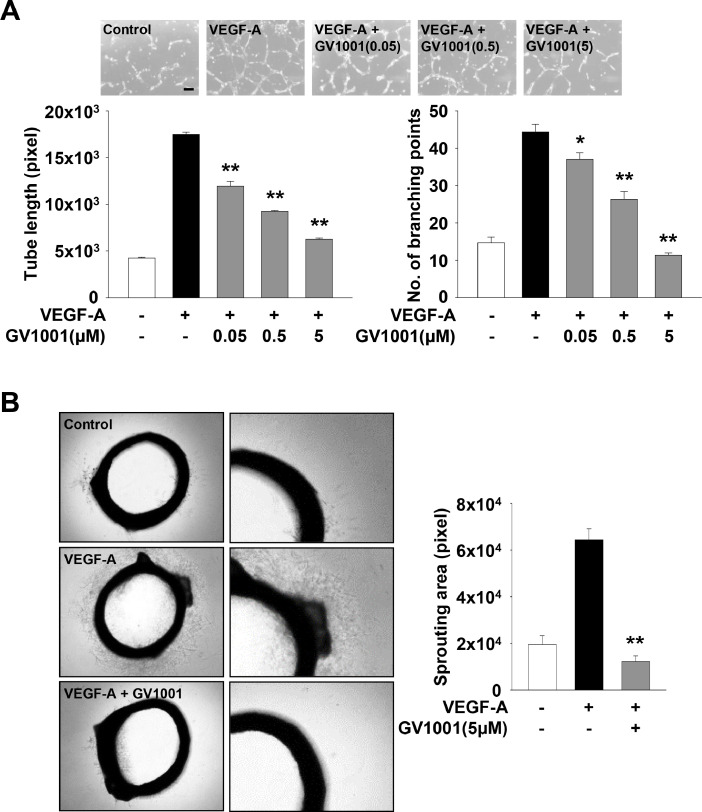
GV1001 inhibits VEGF-A-stimulated tube formation and angiogenic sprouting *ex vivo*. Quiescent cells were treated with GV1001 (0.05–5 μM) for 30 min, followed by VEGF-A (10 ng/mL) stimulation for 6 h. (A) Tube formation results from at least three independent experiments (mean ± SD) are presented as the lengths of tubes (left panel) and the numbers of branching points (right panel) per unit area. Scale bar represents 100 μm. (B) Rat aortic ring assay was performed as described in Materials and methods. Values represent the mean ± SD of at least three independent experiments. Statistical significance is indicated (*p < 0.05, **p < 0.01, compared with VEGF-A-treated cells).

In addition, GV1001 treatment markedly inhibited VEGF-A- or FGF-2-stimulated cell invasion (Fig. 4A, Supplementary Fig. 2B). It has been reported that expression and activation of matrix metalloproteinases (MMPs) induce cell migration and invasion by proteolytic degradation of extracellular matrix (ECM) and cell surface molecules [43,44]. Based on GV1001-mediated inhibition of cell migration and invasion (Figs. 3A and 4A), we next analyzed the change of MMP-2 activity and expression. As shown in Fig. 4B and C, GV1001 treatment suppressed the activity and expression of MMP-2 in VEGF-A-treated HUVECs. Although it cannot be excluded the possibility that GV1001-mediated suppression of cell invasion is partly associated with regulation of other MMPs or tissue inhibitors of metalloproteinases, these results indicate that anti-migratory and anti-invasive activities of GV1001 might be mediated, at least in part, through regulation of expression and proteolytic activity of MMP-2. Moreover, GV1001 significantly abrogated VEGF-A- or FGF-2-stimulated tube formation and microvessel sprouting from rat aortic rings (Fig. 5, Supplementary Fig. 2C). Collectively, these findings show therapeutic potential of GV1001 in regulating angiogenic factor-stimulated responses *in vitro* and *ex vivo*.

### Anti-angiogenic activities of GV1001 are mediated through inhibition of VEGF-A/VEGFR-2 signaling pathways and VEGFR-2 expression

**Fig. 6:**
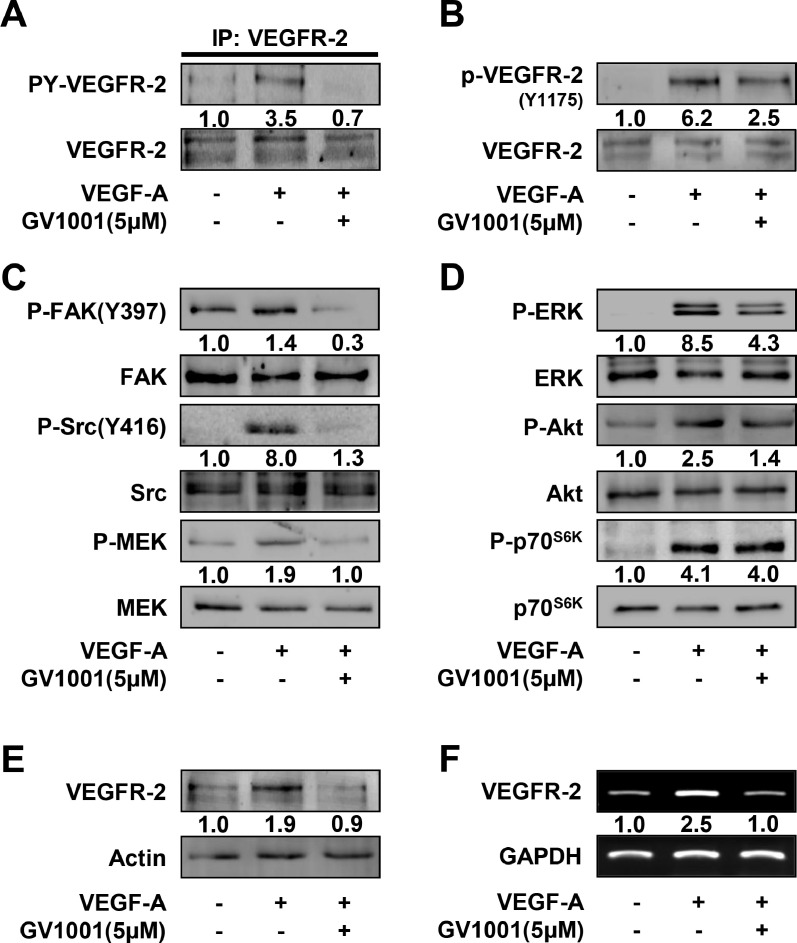
GV1001 regulates VEGF-A-induced signaling pathways and VEGFR-2 expression. Quiescent cells were treated with GV1001 (5 μΜ) for 30 min, followed by VEGF-A (10 ng/mL) stimulation for (A - C) 5 min, (D) 15 min or (E, F) 24 h. (A - E) Western blot and (F) RT-PCR analyses were performed as described in Materials and methods. Results are representative of at least three independent experiments.

To elucidate the molecular mechanisms and targets of GV1001 in modulating angiogenic responses, we examined the changes in activation of VEGFR-2 and its downstream signaling pathways including Src kinase, focal adhesion kinase (FAK), mitogen/extracellular signal-regulated kinase (MEK), p70 S6 kinase (p70^S6K^), ERK and Akt in GV1001-treated HUVECs. As shown in Fig. 6A, GV1001 treatment abrogated VEGF-A-induced phosphorylation of VEGFR-2 on tyrosine residues, which is crucial for initiating downstream signaling pathways involved in angiogenic responses [9,45]. GV1001 blocked the phosphorylation of VEGFR-2 on tyrosine 1175 residue and downstream signaling components including FAK, Src, MEK, Akt and ERK, but not that of p70^S6K^ (Fig. 6B, C and D). Similarly, GV1001 inhibited FGF-2-stimulated phosphorylation of Src and ERK (Supplementary Fig. 2D). Moreover, GV1001 treatment markedly suppressed VEGF-A-induced expression of VEGFR-2, similar to that of untreated controls (Fig. 6E and F). Collectively, these observations indicate that GV1001-mediated inhibition of VEGFR-2 signaling pathways and VEGFR-2 expression may contribute to the regulatory effects of GV1001 on endothelial cell responses and angiogenic sprouting.

### GV1001 exerts anti-cancer activity against NSCLC cells and inhibits the expression and release of VEGF from A549 cells

**Fig. 7:**
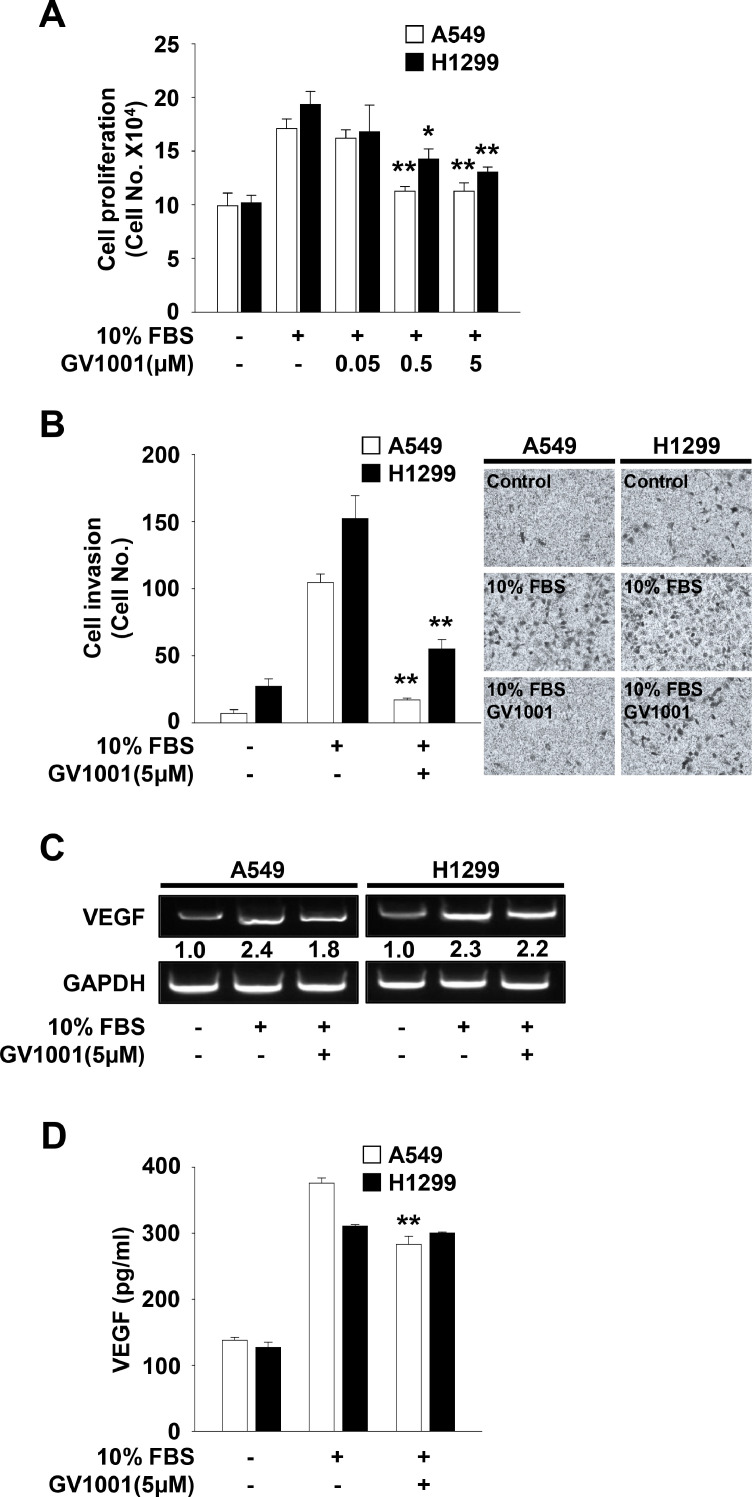
GV1001 has anti-tumor activity against A549 and H1299 cells. Quiescent cells were treated with GV1001 (0.05–5 μM) for 30 min, followed by 10% FBS stimulation for (A, C) 24 h, (B) 18 h or (D) 48 h. (A) The results from at least three independent experiments (mean ± SD) are presented as the fold-increase of untreated controls. (B) Results from at least three independent experiments (mean ± SD) are presented as the numbers of invasive cells. (C) RT-PCR analysis was performed as described in Materials and methods. Results are representative of at least three independent experiments. (D) ELISA assay was performed using conditioned media from cell culture treated as described above. Values represent the mean ± SD of at least three independent experiments. Statistical significance is indicated (*p < 0.05, **p < 0.01, compared with 10% FBS-treated cells).

Based on inhibitory effects of GV1001 on endothelial cell responses, we next investigated the effects of GV1001 on mitogenic responses and production of VEGF in NSCLC cells (Fig. 7A and B). GV1001 treatment dose-dependently inhibited mitogen-stimulated proliferation of p53 wild-type A549 and p53-deficient H1299 cells (Fig. 7A). A549 cells were more sensitive to GV1001-mediated inhibition of cell proliferation, as compared with H1299 cells. Similarly, GV1001-mediated inhibition of invasion in A549 cells appeared to be more potent than that in H1299 cells (Fig. 7B), indicating that GV1001-mediated inhibition of cell proliferation and invasion might be dependent on p53 protein levels. In addition to direct anti-proliferative and anti-invasive activities, GV1001 treatment suppressed mitogen-induced expression and secretion of VEGF in A59 cells, but not in H1299 cells (Fig. 7C and D), similar to previous reports that GV1001 suppresses the expression of VEGF in renal and prostate cancer cells [22,46]. Although the types and levels of molecules secreted from GV1001-treated NSCLC cells remain to be identified, these observations suggest that GV1001-mediated inhibition of soluble factors secreted from cancer cells may regulate tumor-derived angiogenic responses.

## Discussion

VEGF-A/VEGFR-2 signaling pathways play important roles in pathological conditions associated with cancer, inflammatory and ocular diseases as well as physiological angiogenesis [1,6,[47], [48], [49]]. VEGF-A-induced phosphorylation of VEGFR-2 drives the activation of various downstream signaling pathways, which regulates endothelial cell permeability, proliferation, migration and survival. These VEGF-mediated signaling events are coordinately regulated by interactions with VEGFR-2 and other cell surface molecules such as neuropilins, integrins and VE-cadherin [45,50]. Further understanding of VEGF-mediated crosstalk may direct the development of more effective therapeutic strategies and agents to overcome resistance to anti-VEGF/VEGFR therapy.

In the current study, we report that GV1001, a 16-mer peptide within E^611^ and K^626^ of the hTERT, regulates VEGF-A-induced *in vitro* endothelial cell responses including permeability, proliferation, migration, invasion and capillary-like structure formation as well as *ex vivo* angiogenesis. We demonstrate that anti-angiogenic effects of GV1001 involves inhibition of VEGF-A/VEGFR-2 downstream signaling pathways including FAK, Src, MEK, ERK and Akt, redistribution of VE-cadherin to cell–cell contacts, and regulation of cell cycle-related proteins, VEGFR-2 and MMP-2. In addition, our initial experiments indicate that blockade of cyclic AMP production partially abrogates the ability of GV1001 to inhibit VEGF-A-induced cell proliferation, demonstrating the anti-proliferative effect of GV1001 is, at least in part, associated with G protein-coupled receptor (GPCR)-dependent mechanism (data not shown). Although the detailed mechanisms remain to be demonstrated, these findings suggest that regulatory roles of GV1001 in endothelial cell responses might be mediated via binding to GPCRs including gonadotropin-releasing hormone receptor and/or cell-penetrating activity [23,[51], [52], [53]].

In addition to anti-angiogenic activities of GV1001, our findings show that GV1001 exerts anti-proliferative and anti-invasive activities against both p53-positive and p53-deficient NSCLC cells. However, GV1001 inhibits the expression and release of VEGF in p53 wild-type A549 cells only, but not in p53-deficient H1299 cells, suggesting the possibility of p53 involvement in GV1001-mediated differential regulation of VEGF expression and secretion. Although the roles and action mechanisms of GV1001 within the tumor microenvironment consisting of stromal cells and ECM molecules need to be further determined, our observations demonstrate the indirect activity of GV1001 in the regulation of tumor-derived angiogenic responses as well as direct anti-angiogenic and anti-tumor activities.

In conclusion, our results provide significant insights into the regulatory roles and therapeutic efficacy of GV1001 in angiogenesis and cancer progression. Further understanding of the molecular mechanisms and targets in GV1001 anti-angiogenic responses are warranted for the treatment and prevention of a wide range of pathophysiologic angiogenesis-related diseases including cancer.

## Funding

This work was supported by the Basic Science Research Program through the National Research Foundation of Korea funded by the 10.13039/100009950Ministry of Education (2016R1D1A1B04934389) and Ministry of Science and ICT (2021R1F1A1062697), and the Research-Focused Department Promotion Project through the University Innovation Support Program 2020 to Dankook University.

## CRediT authorship contribution statement

**Jae Hyeon Kim:** Conceptualization, Investigation, Methodology, Visualization, Validation, Writing – original draft, Writing – review & editing. **Young-Rak Cho:** Conceptualization, Investigation, Data curation. **Eun-Kyung Ahn:** Conceptualization, Investigation, Data curation. **Sunho Kim:** Investigation, Data curation, Formal analysis. **Surim Han:** Investigation, Data curation, Formal analysis. **Sung Joon Kim:** Investigation, Data curation. **Gyu-Un Bae:** Conceptualization, Resources. **Joa Sub Oh:** Conceptualization, Resources. **Dong-Wan Seo:** Conceptualization, Funding acquisition, Supervision, Resources, Data curation, Writing – original draft, Writing – review & editing.

## Declaration of Competing Interest

The authors declare that there are no conflicts of interest.

## Appendix Supplementary materials

Image, application 1
