# Supplementary material for: A novel telomerase-derived peptide GV1001-mediated inhibition of angiogenesis: Regulation of VEGF/VEGFR-2 signaling pathways
Source: Transl Oncol. 2022 Sep 29;26:101546. doi: 10.1016/j.tranon.2022.101546 (PMC9526227; doi:10.1016/j.tranon.2022.101546)
Supplement: Supplementary file 1 [file mmc1.pdf]

Supplementary Figure 1. Kim et al.

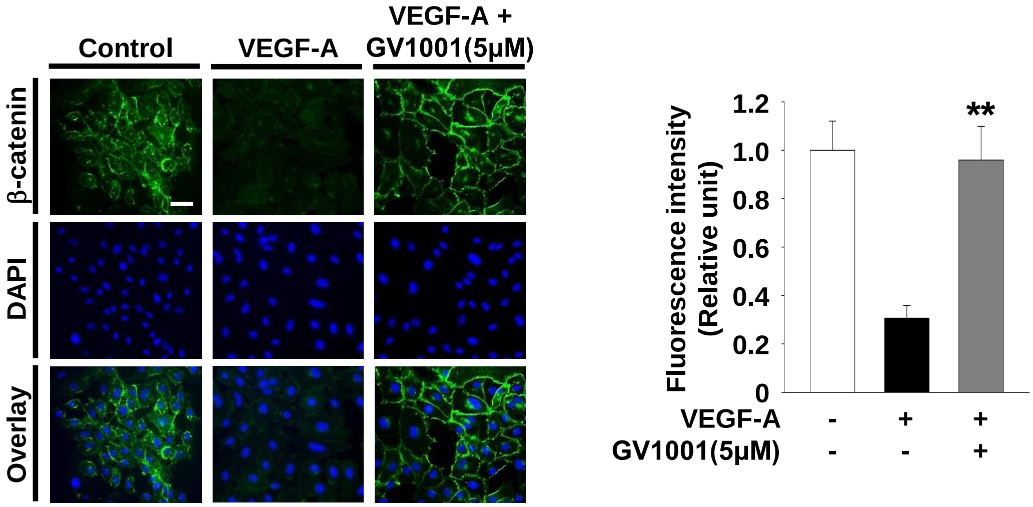

Supplementary Figure 2. Kim et al.

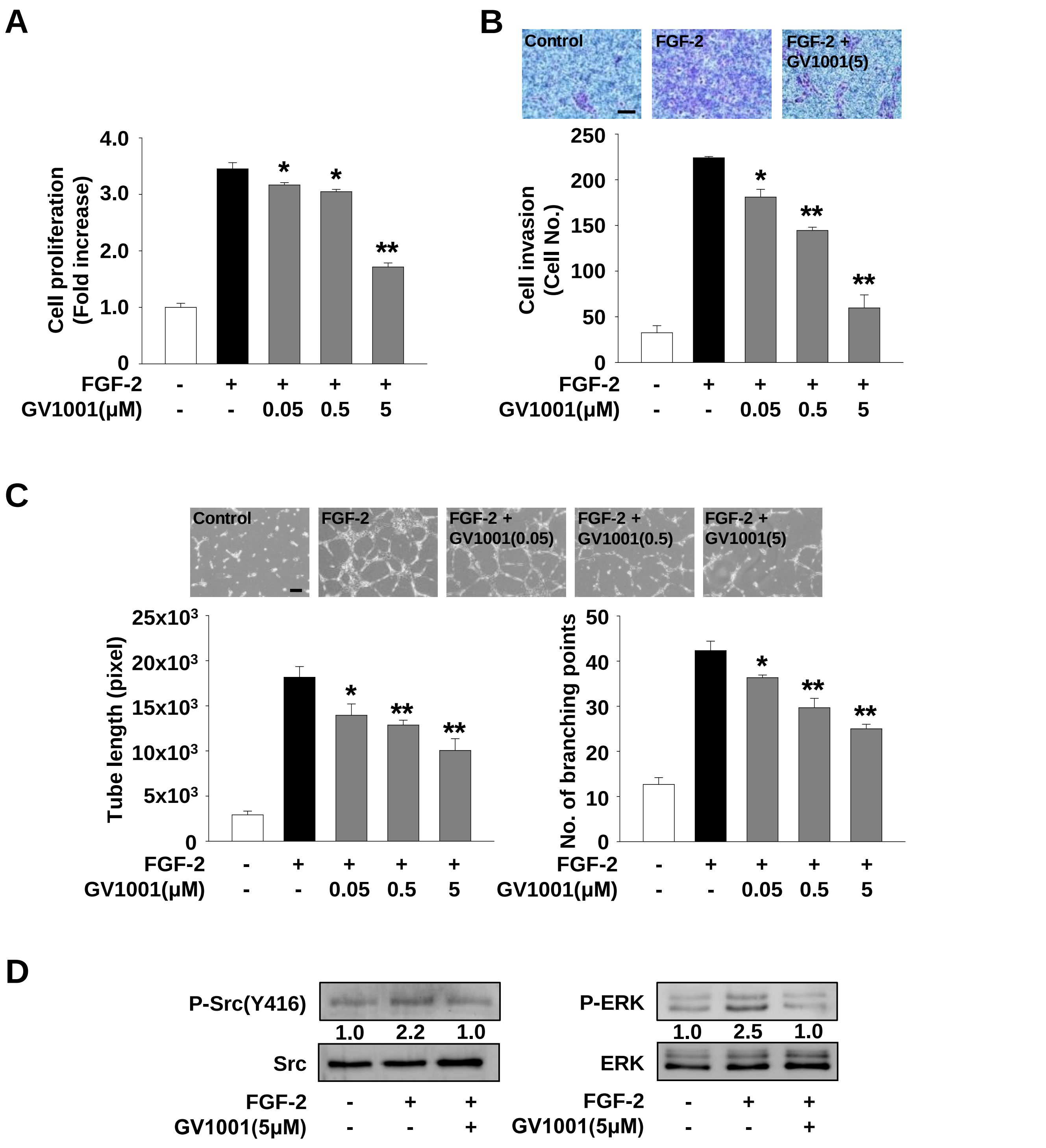

Supplementary figure legends

Fig. S1. GV1001 inhibits VEGF-A-induced loss of β-catenin from cell surfaces.

Quiescent cells were treated with GV1001 (5 μM) for 30 min, followed by VEGF-A (10 ng/mL) stimulation for 30 min. Distribution of β-catenin was determined as described in Materials and methods. DNA was stained with 4’,6-diamidino-2-phenylindole (DAPI). Scale bar represents 10 μm. Statistical significance is indicated (**$p < 0.01$, compared with VEGF-A-treated cells).

Fig. S2. GV1001 inhibits FGF-2-stimulated endothelial cell proliferation, invasion and tube formation.

Quiescent cells were pretreated with GV1001 (0.05 - 5 μM) for 30 min, followed by FGF-2 (50 ng/mL) stimulation for (A) 24 h, (B) 18 h, (C) 6 h or (D) 5 (left panel) and 15 min (right panel). (A) Cell proliferation, (B) *in vitro* transwell invasion, (C) tube formation and (D) Western blot analyses were performed as described in Materials and methods. Results from at least three independent experiments (mean ± SD) are presented as (A) the fold-increase of untreated controls, (B) the numbers of invasive cells or (C) the lengths of tubes (left panel) and the numbers of branching points (right panel) per unit area. Scale bar represents 100 μm. Statistical significance is indicated (*$p < 0.05$, **$p < 0.01$, compared with FGF-2-treated cells). (D) Results are representative of at least three independent experiments.
